# Analysis of the Effect of Radiotherapy on Malignant Pleural Mesothelioma when Given on Adjuvant or Palliative Basis

**DOI:** 10.3779/j.issn.1009-3419.2010.01.10

**Published:** 2010-01-20

**Authors:** Hesham A. El HOSSIENY, Fatma ABOULKSEM, Abdel RHMAN M

**Affiliations:** 1 Department of Radiation Oncology, National Cancer Institute, Cairo, Egypt; 2 Department of Medical Oncology, National Cancer Institute, Cairo, Egypt; 3 Department of Surgery, National Cancer Institute, Cairo, Egypt

**Keywords:** Maligant, Pleural, Mesothelioma, Radiotherapy

## Abstract

**Background and objective:**

This retrospective study was designed to evaluate the response and survival of malignant pleural mesothelioma to radiotherapy when delivered with surgery and chemotherapy and when delivered alone or with chemotherapy.

**Methods:**

A study for 110 patients with malignant pleural mesothelioma who presented to radiotherapy department, National Cancer Institute, Cairo and received radiation therapy in the period from January 1999 to July 2007.

**Results:**

Forty-six patients (41.8%) received trimodality therapy (surgery & adjuvant or neoadjuvant chemotherapy & adjuvant radiotherapy), while bimodality therapy (chemotherapy & radiotherapy) in 38 patients (34.5%), while 26 patients (23.6%) received single modality therapy (palliative radiotherapy), 22 patients (20%) developed local recurrence, 22 patients (20%) developed distant metastases months, 14 patients (12.7%) developed local disease progression, 25 patients (22.7%) are still alive and free of disease at time of reporting. The median survival for all patients was 16 months, while 12 and 18 months overall survival were 63.6% & 31.8% respectively while median survival for stage Ⅱ, Ⅲ, Ⅳ patients was 16.5, 12.5 and 8 months respectively.

**Conclusion:**

Multimodality approach involving surgery, chemotherapy and radiotherapy have been evaluated and proved its superiority in improving survival, especially in stages Ⅱ.

Malignant pleural mesothlioma (MPM) is considered as an aggressive disease with dismal prognosis especially in its diffuse form. Lot of difficulties in accurate diagnosis and staging and even in its treatment contributed actively to this dismal prognosis^[1]^, its etiology is related to asbestos fibers exposure especially the blue crocidolite and is characterized by a long latency period^[2]^, its incidence is increasing in western countries and in countries with poor regulations of asbestos mining, industrial production and house hold use^[3]^.

Reports of National Cancer Institute Cairo University showed increased relative frequency of MPM in the last few years^[4]^.

Single modality therapy has failed in significantly changing the natural history of the disease and its median survival, except in early localized disease which could be completely resected by surgery without the need of adjuvant therapy^[5]^.

Multimodality aggressive therapy which include extensive surgical resection of the disease up to extrapleuralpneumonectomy (EPP) to be followed by radiotherapy and chemotherapy (pre or postoperative chemotherapy) had proved to increase median survival especially in stages Ⅱ and Ⅲ^[6, 7]^.

The use of radiotherapy for MPM faces many difficulties including a very large target volume to be covered and also the need of high tumercidal dose which when given it could damage the surrounding normal tissues including lung, spinal cord, heart *etc*, thus the use of radiation as a single radical modality therapy is not possible as the tolerance of the lung is 20 Gy with the V20 of the contralateral lung not exceeding 20 Gy, mean liver dose not exceeding 30 Gy, spinal cord 45 Gy (more than 10 cm segment), 70% of the heart should receive less than 45 Gy while oesophagus 45 Gy-50 Gy^[8]^.

There are several approaches to integrating postoperative radiotherapy into the trimodality program. The lowest locoregional recurrence rates post-EPP are in those series using highdose postoperative hemithorax Irradiation^[9]^.

Surgery in an attempt for aggressive debulking and cytoreduction can be either pleurectomy-decortication (P/D) or EPP. Each of there procedures has no major effect on survival in diffuse type and more treatment is needed, so adjuvant chemoradiotherapy was attempted in selected patients who can tolerate such aggressive therapy regarding their organ functions especially lung, heart, renal and hepatic functions.

Single agent and combination chemotherapy have been evaluated in single and combined modality studies. The most studied agent is doxorubicin, which has produced partial responses in approximate 15%-20%^[10]^.

Some combination chemotherapy regimens have been reported to have higher response rates in small phase Ⅱ trials. However, the toxicity reported is also higher and there is no evidence that combination regimens result in longer survival or longer control of symptoms. Recurrent pleural effusions may be treated with pleural sclerosing procedures; however, failure rates are usually secondary to the bulk of the tumor, which precludes pleural adhesion due to the inability of the lung to fully expand^[11, 12]^.

Byrne *et al*^[13]^ first described a 47% response rate with a combination of cisplatin and gemcitabine, and a follow-up multicenter trial from Australia with 53 patients reported a 26% rate of activity but a median survival of only 7.5 months. The activity of the combination in other multicenter phase Ⅱ studies^[14]^, in patients previously treated with other chemotherapy, and of the gemcitabine/carboplatin regimens^[15]^ has led to its widespread use. Gemcitabine^[16, 17]^, cisplatin and carboplatin all have independent but modest single agent activity.

A novel antifolate, pemetrexed, demonstrated broad antitumor activity in phase Ⅰ and Ⅱ trials^[18]^. When combined with cisplatin, pemetrexed induced regressions in 38% of pleural mesothelioma patients^[19]^.

Another antimetabolite, raltitrexed (Tomudex; an agent not available in many countries), a 240 patient phase Ⅲ trial comparing cisplatin to raltitrexed plus cisplatin has been reported showing that the median survival of patients treated with the doublet was 11.4 months compared to the survival after cisplatin alone of 8.8 months (*P*=0.048)^[20]^.

The aim of this study is to evaluate the effect of radiotherapy when given on adjuvant basis and combined with chemotherapy or when given on palliative basis either alone or combined with chemotherapy.

## Patients and methods

A study for 110 patients with malignant pleural mesothelioma who presented to radiotherapy department in National Cancer Institute, Cairo, and received radiation therapy in the period from January 1999 to July 2007 (Fig 1).

**1:**
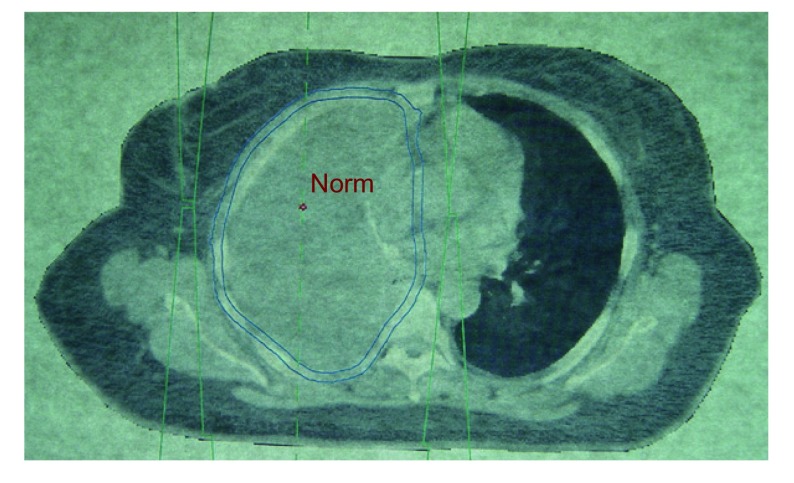
Radiation plane following EPP

Data from patients files were revised regarding stage of disease, pathologic subtype, full details of radiotherapy received, other therapy received (surgery or chemotherapy), response to treatment and survival overall survival was calculated using the *Kaplen-Meier* estimates, while the *Log-rank* test was used for comparing survival curves.

## Results

### The characteristics of patients

The age of patients ranged from 29 to 73 years with median age of 49 years, there were 70 (63.6%) males & 40 (36.4%) females. 95 patients (86%) living in endemic areas (around asbestos factories). 75 patients (68%) were smokers. 32 patients (29%) were industrial workers.

### Clinical picture at presentation

All patients had dyspnea, 80 patients (73%) had chest pain, 90 patients (82%) had cough while 62 patients (56%) had haemoptesis.

### Histopathologic subtypes

Epithelioid subtype was encountered in 70 patients (63.6%), sarcomatoid subtype in 22 patients (20%) while biphasic subtype was in 18 patients (16.4%).

### Stage

The patients were staged according to (Inernational Mesothelioma Interest Group [IMIG]): 60 patients (54.5%) had stage Ⅱ and 30 patients (27.3%) had stage Ⅲ while stage Ⅳ was encountered in 20 patients (18.2%).

Patients who underwent EPP were staged radiologicaly and pathologically (*i.e.* 40 patients), while the rest of the studied patients were staged radiological only.

### Treatment received

Patients were treated with each treatment modality according to their stage of disease and their performance status, so that patients with operable and early stage disease and with good performance were offered the trimodality treatment while those with more advanced stage and poor performance was offered either the bimodality or the single modality treatment.

Forty six patients (41.8%) received trimodality (adjuvant) therapy (surgery and adjuvant or neoadjuvant chemotherapy & adjuvant radiotherapy which started at 6 weeks median duration following surgery), all of them received 50 Gy/25 f /5 w (all patients were stage Ⅱ, *i.e.* 77% of stage Ⅱ patients received trimodality therapy), while bimodality (palliative) therapy (chemotherapy & radiotherapy) in 38 patients (34.5%), (25 patients of them received 50 Gy/25 f/5 w while 13 patients received 40 Gy/20 f /4 w) (24 patients of them were stage Ⅲ & 14 were stage Ⅱ, all of them were treated on palliative basis, 22 patients of them were treated to prevent skin metastases), while 26 patients (23.6%) received single modality (palliative) therapy (palliative radiotherapy) 15 patients of them received 30 Gy/10 f/2 w and 11 patients received 500 Gy/4 f/1 w, (6 patients of them were stage Ⅲ & 20 patients were stage Ⅳ, all of them were treated on palliative basis, 15 patients of them were treated to prevent skin metastases).

Forty patients (36.4%) underwent EPP and all of them achieved negative surgical margin, while 6 patients underwent pleural decortication.

Eighty four patients (76.4%) received chemotherapy either neoadjuvant (30 patients) or adjuvant (16 patients), 38 patients received chemotherapy with radiotherapy (28 patients of them received chemotherapy before radiotherapy, while 10 patients received chemotherapy after radiotherapy), different drug regimens were used 67 patients received gemcitabine and cisplatin or carboplatin while the remaining patients received different protocols including vepsid or navelbine or adriamycin or alemta, patients typically received 4-6 cycles.

Thirty patients (27.3%) received photon beam only, 70 patients (63.6%) received combined photon and electrone beams, while 10 patients (8%) received electron beam only (for palliation). Regarding field extent 90 patients (81.8%) received hemithoracic fields, while 20 patients (23%) received localized fields, 92 patients (83.6%) received 2 parallel opposing fields, 13 patients (11.8%) received direct field using either photon or electron beam fields, 4 patients (3.6%) received 2 wedged oblique fields while 1 patient (0.9%) received 3D conformal hemithoracic radiotherapy.

### Response to treatment

Response to treatment were evaluated 2-3 weeks after end of radiotherapy using CT chest and abdomen and clinical examination also by recording the patients complaints of pain, dyspnea, cough, *etc*.

Twenty five patients developed complete response through EPP & chemoradiotherapy, while 20 patients developed temporary partial response which lasted for 3-5 months.

Twenty seven patients (24.5%) have no response regarding tumor size, dyspnea, chest pain & cough, of them (17 patients were stage Ⅱ, 6 patients stage Ⅲ & 6 patients were stage Ⅳ, 4 patient was biphasic subtype & 25 patients were epetheliod subtype, 21 patients received 50 Gy/25 f /5 w & 4 patients received 40 Gy/20 f/4 w & 4 patient received 30 Gy/10 f/2 w, while 17 patients did not receive chemotherapy).

Twenty two patients (20%) developed local recurrence after an average duration of 8 months after end of treatment, of them (6 patients underwent P/D & 16 patients underwent EPP while 18 patients of them received chemotherapy, 17 of them were stage Ⅱ & 5 patients was stage Ⅲ, while all patients received adjuvant radiotherapy to hemithorax at a dose of 50 Gy/25 f/5 w), 15 patients developed local recurrence intrathoracically (at operation side) while 7 patient developed in addition chest wall skin nodules).

Twenty two patients (20%) developed distant metastases after an average duration of 7 months after end of treatment of them (7 patients with liver mets. and malignant ascites, 10 patients developed bone metatases & 5 patients with liver and bone mets, 15 patients of them received chemotherapy, 14 patients of them were in the single modality group while 8 patients were in the bimodality group these data was at the time of reporting).

Fourteen patients (12.7%) developed local disease progression with increasing in tumor size, pleural effusion and dyspnea, chest pain & cough after an average duration of 5 months after end of treatment of them (10 patients were stage Ⅳ & 4 patients was stage Ⅲ, all of them underwent pleural biopsy only, 8 patients did not receive chemotherapy, while all of them received hemithoracic irradiation at a dose of 30 Gy-50 Gy).

Twenty-five patients (22.7%) are still alive till time of reporting and free of disease, (23 of them underwent EPP & 2 of them underwent pleural decortication, 15 patients of them received neoadjuvant chemotherapy, all of them received adjuvant radiotherapy at a dose of 50 Gy/25 f/5 w, 23 patients of them were stage Ⅱ and 2 of them were stage Ⅲ).

Patients who received radiotherapy only (26 patients) had improved chest pain in 15 patients of them (57.7%).

Median survival for all patients was 16 months, while 12 months & 18 months overall survival rates were 63.6% & 31.8% respectively (Fig 2, Tab 1).

**2:**
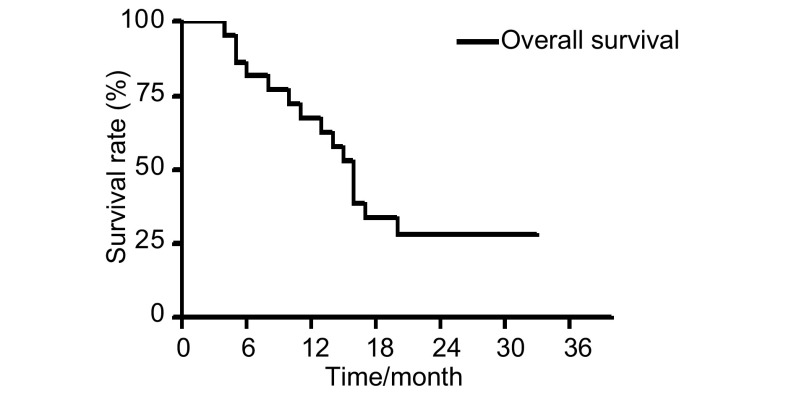
Overall survival of all studied patients

**1:** Median survival by stage of disease: *P*=0.000 12

Stage of disease	Median Survival (month)
Ⅱ	16.5
Ⅲ	12.5
Ⅳ	8.0

Comparing overall survival by different stages of disease, it showed that 18 months overall survival for stages Ⅱ, Ⅲ & Ⅳ were 41.6%, 33.3% & 0% respectively, which was statistically significant (*P*= 0.000 12)(Fig 3, Tab 2, 3).

**3:**
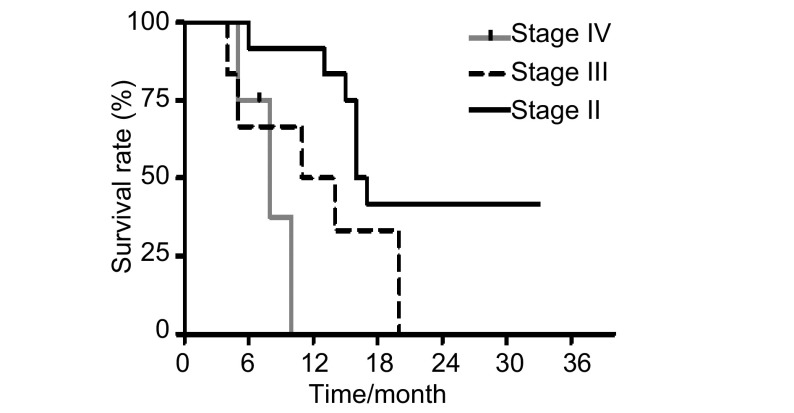
Overall survival by stage of disease

**2:** Median survival by pathologic subtype: *P*=0.36

Pathologic subtype	Median survival (month)
Epitheloid subtype	17 (4-37)
Biphasic & sarcomatoid subtypes	13 (2-29)

**3:** Median survival by treatment modality

Treatment modality	Median survival (month)
Trimodality (surgery+Rth+Cth)	16
Bimodality (Cth + Rth)	11
Singlemodality (Rth palliative)	8

## Discussion

Results of radiotherapy for malignant pleural mesothelioma have been generally disappointing, doses below 30 Gy have produced only temporary relief of symptoms in some cases, and doses in excess of 40 Gy are needed to achieve adequate palliation, with photon alone or combined with electron beam then followed by a boost (localized to residual tumor) to a dose of 60 Gy-70 Gy^[21, 22]^. Higher doses to a larger volume can produce significant complications such as radiation pneumonitis, myelitis and hepatitis which could be fatal^[21]^.

Recent guidelines for three-dimensional conformal radio-therapy suggest a dose of 54 Gy in 30 fractions five days per week to the ipsilateral thoracic cavity, chest wall incisions, and drains, with attention to normal tissue tolerance for the contralateral lung, spinal cord, heart, esophagus, and other vital structures^[23]^.

Intensity-modulated radiotherapy (IMRT) is a promising newer technology that may deliver better local control results^[24]^; however it is not widely available, and there have been reports of subsequent fatal pneumonitis which suggest caution in implementing this technology^[25-27]^.

Nevertheless, only patient series using an aggressive multimodality approach achieve clinically meaningful five-year survival rates^[28, 29]^.

Radiation therapy is used effectively to prevent seedling in the biopsy track and open biopsy scar by using a dose of 21 Gy over 3 fractions, as it decreases the incidence of wound implants by malignant cells from 60% to less than 5%^[30, 31]^.

Malignant pleural mesothelioma is a challenging disease in all of its aspects either at presentation, diagnosis, staging or treatment.

In comparison with Calavrezos *et al*^[32]^, and Sugarbaker *et al*^[29]^, who reported that the median survival in patients who received trimodality treatment was 13 and 17 months respectively, which is comparable to this study that the median survival for patients who received trimodality treatment in this study was 16 months.

Malignant seeding in approximately 20% to 50% of mesothelioma patients along thoracentesis tracts, biopsy tracts, chest tube sites, and surgical incisions is a common complication of procedures in these patients, 40 patients were randomised after an invasive diagnostic procedure to either RT or no treatment. No patient in the radiation treatment group developed subcutaneous nodules. Alternatively, 8 of 20 patients in the untreated group developed metastases^[33]^.

These results also compared favorably with the series from MD Anderson with a median survival after neoadjuvant chemotherapy which was followed with extrapleural pneumonectomy followed by adjuvant intensity modulated radiotherapy which showed a median survival of 15 months^[34]^.

Also in comparison to El-Shafiey MM^[4]^, it showed a median survival of 9 months for patients who received bimodality treatment (RTH+CTH) while in this study it showed 11 months median survival, also our study showed comparable median survival regarding patients who received radiotherapy only, this is attributed to that patients in both studies were with advanced or recurrent disease and with poor performance status which results in poor radiation response.

Also it was found that palliative radiotherapy as a single modality can improve pain in around 60% of patients^[35]^, but the effect is generally short-lived, this was in concordance with this study were (57.7%) of patients who received palliative radiotherapy had improved chest pain.

## Conclusions

Single modality therapy was the initial approach to this disease, its generally has not been effective in changing natural history of the disease. Multimodality approach involving surgery, chemotherapy & radiotherapy have been evaluated and proved its superiority in improving survival especially in stages Ⅱ, but still with low survival rates which results in the needs to explore for newer treatment strategies.

### Acknowledgement

Fatma Aboulkasem, Abdel Rahman M., both of them helped the main auther in collecting patients data.

### Conflict of interest statement

There is no conflict of interest (Non declared).
